# The ‘forma specialis’ issue in *Fusarium*: A case study in *Fusarium solani* f. sp. *pisi*

**DOI:** 10.1038/s41598-018-19779-z

**Published:** 2018-01-19

**Authors:** Adnan Šišić, Jelena Baćanović-Šišić, Abdullah M. S. Al-Hatmi, Petr Karlovsky, Sarah A. Ahmed, Wolfgang Maier, G. Sybren de Hoog, Maria R. Finckh

**Affiliations:** 1 Department of Ecological Plant Protection, University of Kassel, Witzenhausen, Germany; 2 Department of Organic Plant Breeding and Agrobiodiversity, University of Kassel, Witzenhausen, Germany; 3 Westerdijk Fungal Biodiversity Institute, Utrecht, The Netherlands; 4 Centre of Expertise in Mycology Radboud University Medical Centre/ Canisius Wilhelmina Hospital, Nijmegen, The Netherlands; 5 Directorate General of Health Services, Ministry of Health, Ibri Hospital, Ibri, Oman; 6 Department of Molecular Phytopathology and Mycotoxin Research, University of Göttingen, Göttingen, Germany; 7 Faculty of Medical Laboratory Sciences, University of Khartoum, Khartoum, Sudan; 8 Julius Kühn-Institut, Federal Research Centre for Cultivated Plants, Institute for Epidemiology and Pathogen Diagnostics, Braunschweig, Germany; 9 Basic Pathology Department, Federal University of Paraná State, Curitiba, Paraná Brazil

## Abstract

The *Fusarium solani* species complex (FSSC) has been studied intensively but its association with legumes, particularly under European agro-climatic conditions, is still poorly understood. In the present study, we investigated phylogenetic relationships and aggressiveness of 79 isolates of the FSSC collected from pea, subterranean clover, white clover and winter vetch grown under diverse agro-climatic and soil conditions within Temperate and Mediterranean Europe. The isolates were characterized by sequencing *tef1* and *rpb2* loci and by greenhouse aggressiveness assays. The majority of the isolates belonged to two lineages: the *F*. *pisi* comb. nov. lineage (formerly *F*. *solani* f. sp. *pisi*) mainly accommodating German and Swiss isolates, and the *Fusisporium* (*Fusarium*) *solani* lineage accommodating mainly Italian isolates. Based on the results of aggressiveness tests on pea, most of the isolates were classified as weakly to moderately aggressive. In addition, using one model strain, 62 accessions of 10 legume genera were evaluated for their potential to host *F*. *pisi*, the species known mainly as a pathogen of pea. A total of 58 accessions were colonized, with 25 of these being asymptomatic hosts. These results suggest a broad host range for *F*. *pisi* and challenge the forma specialis naming system in *Fusarium*.

## Introduction

*Fusarium solani* (sexual morph *Nectria haematococca*; syn. *Haematonectria haematococca*) is a filamentous fungus of significant agricultural importance that has been accommodated as a single species in the section *Martiella* within the genus *Fusarium*^1^. Re-evaluation of species taxonomy based on molecular phylogenetic analyses has revealed that *F*. *solani* is a species complex (FSSC) which includes at least 60 distinct phylogenetic species^2,3^. Members of the complex are globally distributed and of considerable ecological plasticity, causing infections in both plants and humans^4,5^.

Phytopathogenic species within the FSSC include some of the most economically important plant pathogens associated with vascular wilts and root rots in over 100 crops^6^. Despite the broad host range of the complex as a whole, individual species are often associated with only one or a few plant hosts. Consequently, populations of *F*. *solani* pathogenic on plants have been divided into 12 formae speciales and two races^7–10^.

Early studies on sexual compatibility of special forms and races has already shown that *F*. *solani* represents at least seven biological species classified as mating populations (MPs) I-VII, with *Nectria haematococca* as the most commonly referred sexual morph. Biological species correlated with a host range, as successful sexual crosses were found only among heterothallic isolates within each special form or race^11^. However, the designation forma specialis may lead to incorrect assumptions concerning the aggressiveness and host specificity of individual isolates. For example, studies on the host range of *F*. *solani* f. sp. *pisi* (MP VI), named by its specific pathogenicity on pea (*Pisum sativum*), revealed that the species was also pathogenic on chickpea (*Cicer arietinum*) as well as several other non-legume hosts, such as ginseng (*Panax ginseng*) and mulberry tree (*Morus alba*)^12,13^. Similar results have been reported for the host range and aggressiveness of *F*. *virguliforme* (formerly *F*. *solani* f. sp. *glycines*) and *F*. *solani* f. sp. *eumartii*^14,15^. Thus, the term forma specialis is often misleading and will most likely need to be reconsidered in the future.

Traditional taxonomic methods for identifying special forms and races rely on morphological criteria, aggressiveness tests and sexual compatibility. They are time consuming, labor intensive and often inconclusive. The use of morphological criteria for diagnostic purposes requires extensive knowledge of classical taxonomy and still remains difficult due to overlapping morphological characters among many closely related species. In the case of aggressiveness tests on a specific host plant, the environmental factors and genetic makeup of the host may have significant influence on the bioassay outcome. Similarly, the biological species concept on the basis of sexual crosses in *F*. *solani* has several problems including unequal frequencies of mating type alleles in different populations, failure of compatible isolates to reproduce due to male or female dominance, or simply environmental conditions suppressing sexual reproduction^4^.

In the past 10 to 15 years, molecular phylogenetic approaches have been extensively employed to facilitate accurate species identification in the genus *Fusarium*^3,16,17^. Polymorphisms in DNA sequences of the translation elongation factor 1 alpha (*tef1*) and the second largest subunit of RNA polymerase II (*rpb2*) have provided a robust and reliable means for phylogenetic species recognition within the FSSC and the genus *Fusarium*^18,19^. These protein coding gene regions show a high level of sequence polymorphism among closely related species, do not have non-orthologous copies and can be amplified from all species of the genus using single pairs of universal primers^20^.

The main objectives of this study were to investigate diversity, geographical patterns of host preference and phylogenetic relationships among the FSSC isolates collected from pea, subterranean clover, white clover and winter vetch grown under diverse agro-climatic and soil conditions within Temperate and Mediterranean Europe. To that end, we conducted gene sequence analysis, aggressiveness bioassays and sought to clarify the host range of *F*. *solani* f. sp. *pisi* by determining the potential of various legumes to symptomatically or asymptomatically host the fungus. Based on our results, we propose a taxonomic recombination by assigning isolates of *Fusarium solani* f. sp. *pisi* to *Fusarium pisi* comb. nov. Therefore, in this article the fungus is being referred to as *Fusarium pisi*.

## Results

### Phylogeny

Phylogenetic analyses inferred from the *tef1* and the *rpb2* sequences resolved the phylogenetic positions of the 83 isolates studied (including *F*. *redolens*) in relation to currently recognized monophyletic species in the FSSC complex. The *tef1* data set included 122 strains and consisted of 720 characters including alignment gaps, of which 220 characters were parsimony informative; the *rpb2* data set included 59 strains and consisted of 910 characters with alignment gaps, of which 168 were parsimony informative, and the concatenated *tef1* and *rpb2* gene sequence included 56 strains and comprised 1600 characters including alignment gaps, of which 262 were parsimony informative.

**Figure 1:**
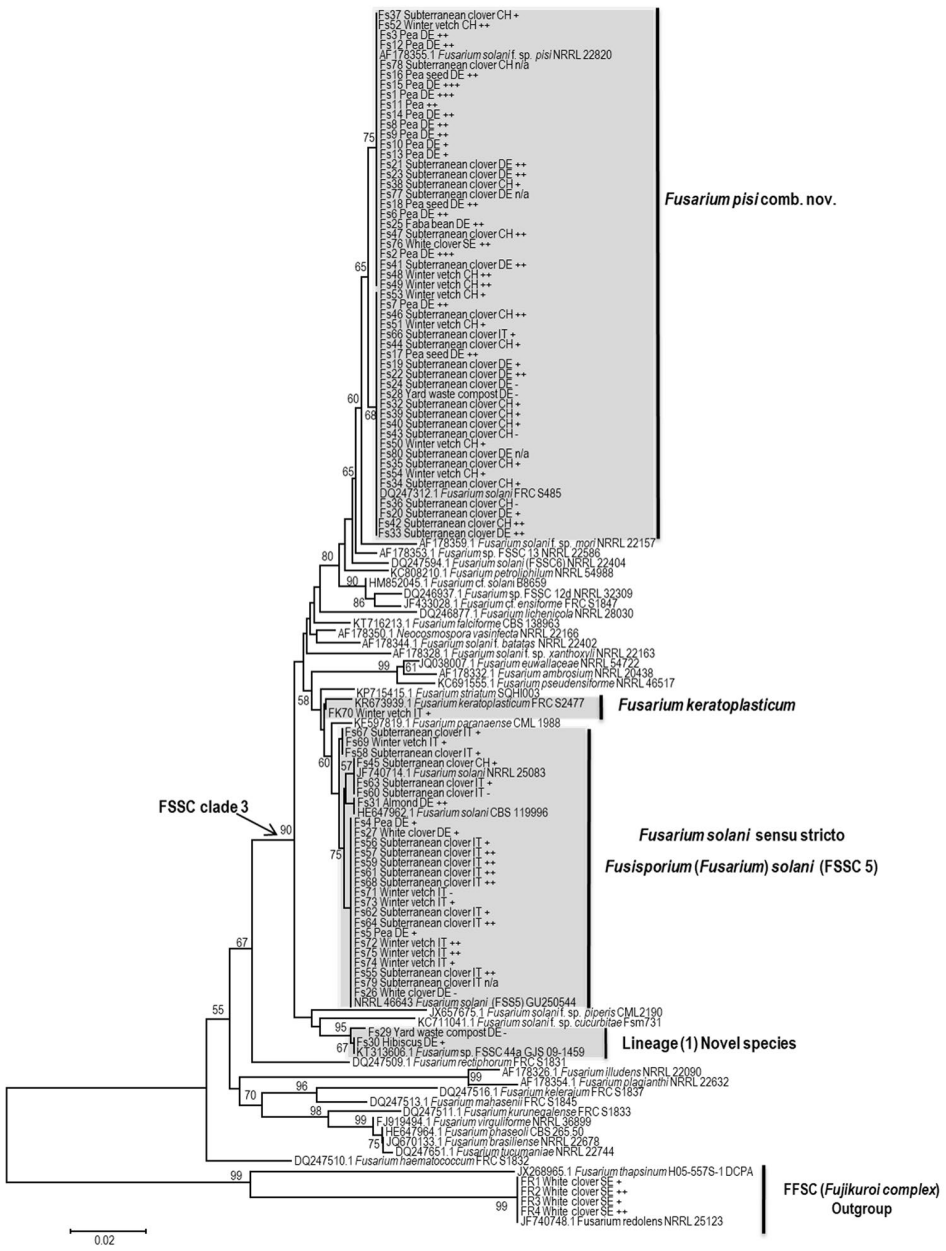
Phylogenetic tree resulting from RAxML analysis for the *tef1* gene sequences. The data set comprised 720 characters with alignment gaps, and included 122 sequences with reference strains. Maximum Likelihood analysis was performed by RAxML with non-parametric bootstrapping using 1000 replications. The tree was rooted with four strains of *F*. *redolens* collected for the purpose of this study together with reference strains *F*. *redolens* NRRL 25123 and *F*. *thapsinum* H05-557S-1DCPA. Isolate ID number is followed by host plant and country of origin, where IT = Italy, CH = Switzerland, DE = Germany, SE = Sweden; Based on the results of the greenhouse experiment 1, the symbols −, +, ++, and +++ indicate non-aggressive, weakly aggressive, moderately aggressive and highly aggressive isolates on pea, respectively; n/a = not included in aggressiveness test.

**Figure 2:**
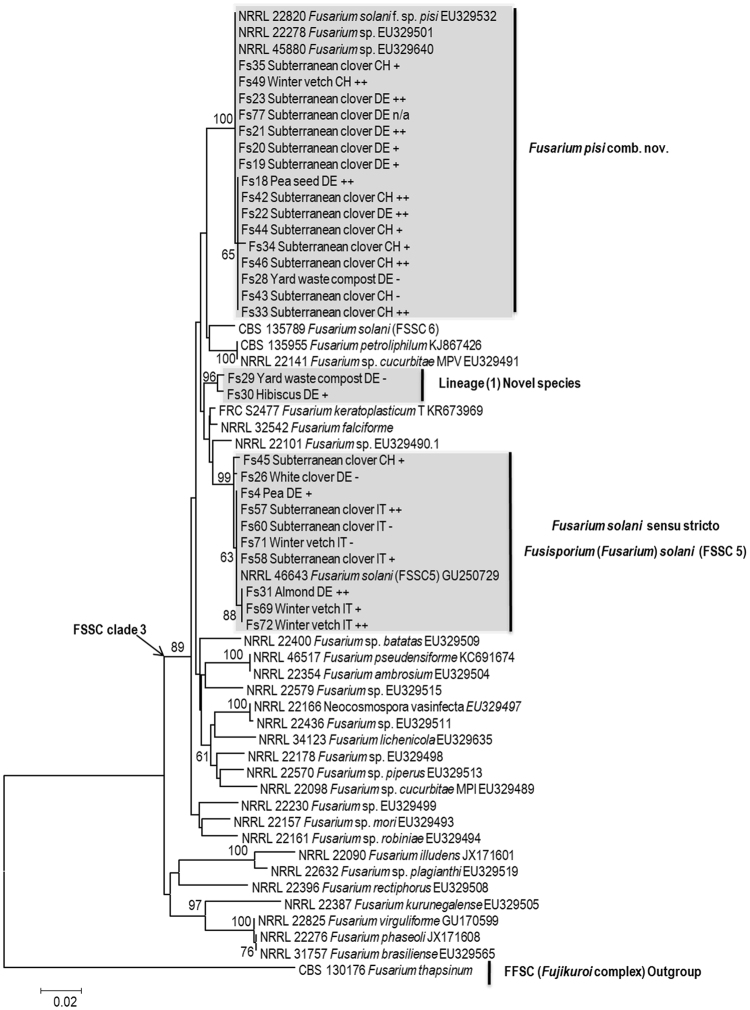
Phylogenetic tree resulting from RAxML analysis for the *rpb2* gene sequences. The data set comprised 910 characters with alignment gaps, and included 56 sequences with reference strains. Maximum Likelihood analysis was performed by RAxML with non-parametric bootstrapping using 1000 replications. The tree was rooted with one strain of *F*. *thapsinum* CBS 130176. Isolate abbreviations are provided in the caption of Fig. 1.

**Figure 3:**
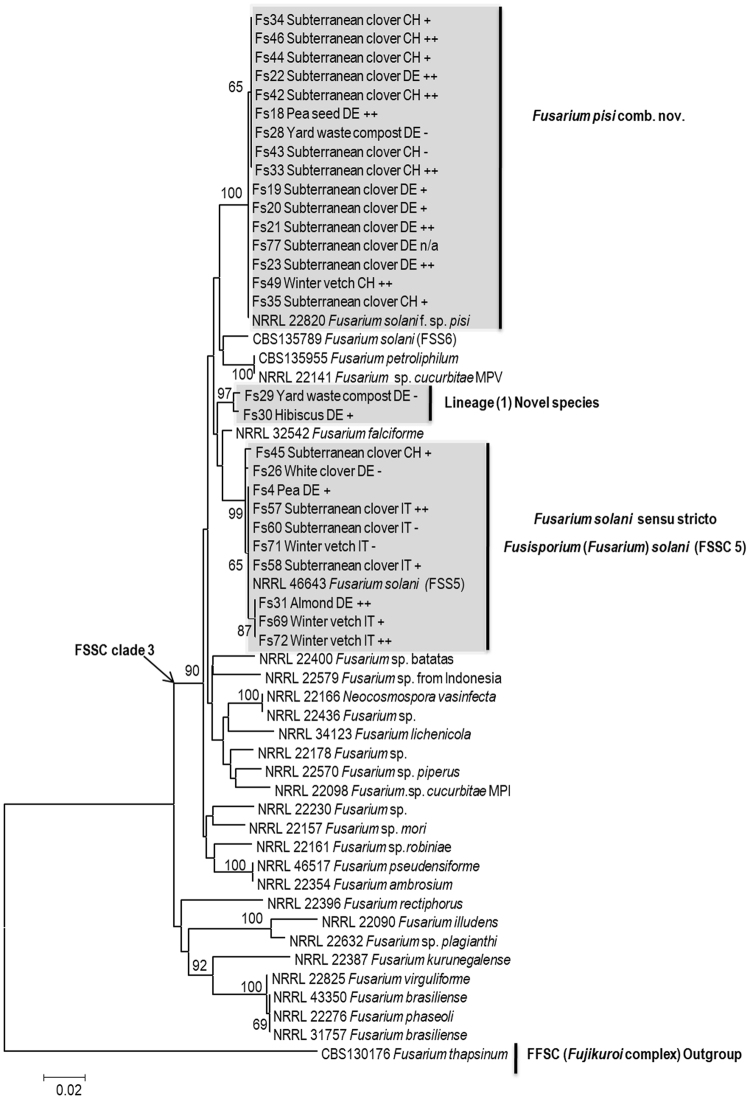
Phylogenetic tree resulting from RAxML analysis for the combined *rpb2* and *tef1* gene sequences. The data set comprised 1600 characters with alignment gaps and included total of 56 sequences with reference strains. Maximum Likelihood analysis was performed by RAxML with non-parametric bootstrapping using 1000 replications. The tree was rooted with one strain of *F*. *thapsinum* CBS 130176. Isolate abbreviations are provided in the caption of Fig. 1.

The isolates studied formed four different lineages, all nested within the FSSC clade 3 (Fig. 1). According to the single locus phylogenetic analysis, based on the *tef1* tree, 51 isolates were placed in two closely related subclades in the *F*. *pisi* lineage: the first group of 27 isolates matched with *F*. *pisi* comb. nov. (NRRL 22820) with a 75% bootstrap value, and the second group of 24 isolates matched with *Fusarium solani* (FRC S485) with a 68% bootstrap value. Based on the tree generated from *rpb2* sequencing with representative isolates from both sub-clades, the topological differences did not receive any additional significant support on either *rpb2* or concatenated *tef1 - rpb2* trees. The same strains that previously formed two sub-clades (*tef1* tree, Fig. 1) were nested in one clade that matched *F*. *pisi* (NRRL 22820) with some strains showing low intraspecific variation (Figs 2 and 3). Thus, based on the phylogenetic network obtained from concatenated gene trees, all 51 strains were assigned to a single species i.e. *F*. *pisi*. With the exception of Fs66 recovered from the roots of subterranean clover grown in Italy and Fs76 recovered from white clover roots grown in Sweden, the strains nested in the *F*. *pisi* lineage originated from different localities in Germany and Switzerland. These strains were recovered from all four legume hosts and included 25 strains from subterranean clover, 7 strains from winter vetch, 16 strains from pea, one strain from faba bean roots and one strain recovered from yard waste compost.

A group of 25 isolates were placed in the *Fusarium solani sensu stricto* (FSSC 5) lineage, recently assigned an epitype specimen by Schroers *et al*.^2^ to define the species *Fusarium solani s*. *str*., which was recombined by the same authors in *Fusisporium* as *Fusisporium solani*. Similar to *F*. *pisi*, the relative distances using the single and the combined gene analyses (two locus tree) revealed intraspecific variation in *Fusisporium* (*Fusarium*) *solani s*. *str*. (Figs 1–3). This lineage primarily accommodated isolates recovered from subterranean clover and winter vetch roots collected in Italy. The few exceptions included six strains collected in Germany and Switzerland, namely two pea, one cherry and two white clover strains from Germany and one subterranean clover strain from Switzerland (Figs 1–3).

The results of the *tef1* tree topology also revealed one strain (FK70) matching with *F*. *keratoplasticum* (FRC S2477, Fig. 1). Strains Fs29 and Fs30, recovered from compost and hibiscus, respectively, did not cluster with any of the currently recognized FSSC species (Figs 1–3). Based on distances from the nearest neighbor species and high bootstrap support values (≥96%) between sub-clusters using both the single and the two gene phylogenetic analyses (*tef1* and the *rpb2* sequences), our results suggest that these two isolates represent at least one new lineage within the FSSC clade 3.

All of the tested isolates were scattered within clade 3 without evidence of phylogenetic structure with respect to host. Four isolates of *F*. *redolens*, recovered from white clover grown in Sweden were placed in the *F*. *fujikuroi* species complex and used as an outgroup for species level resolution within the *tef1* locus.

### Taxonomy

Fusarium pisi (Jones) A. Šišić, J. Baćanović-Šišić, S. A. Ahmed & A. M. S. Al-Hatmi, comb. nov.

=*Fusarium martii* Appel & Wollenw var. *pisi* Jones, *J*. *Agric*. *Res*., Washington **26**: 459 (1923) (basionym).

=*Fusarium solani* (Mart.) Sacc. f. *pisi* (Jones) W.C. Snyder & H.N. Hansen, *Amer*. *J*. *Bot*. 28: 740, 1941. (presently considered as *F*. *solani* f. sp. *pisi*).

**Figure 4:**
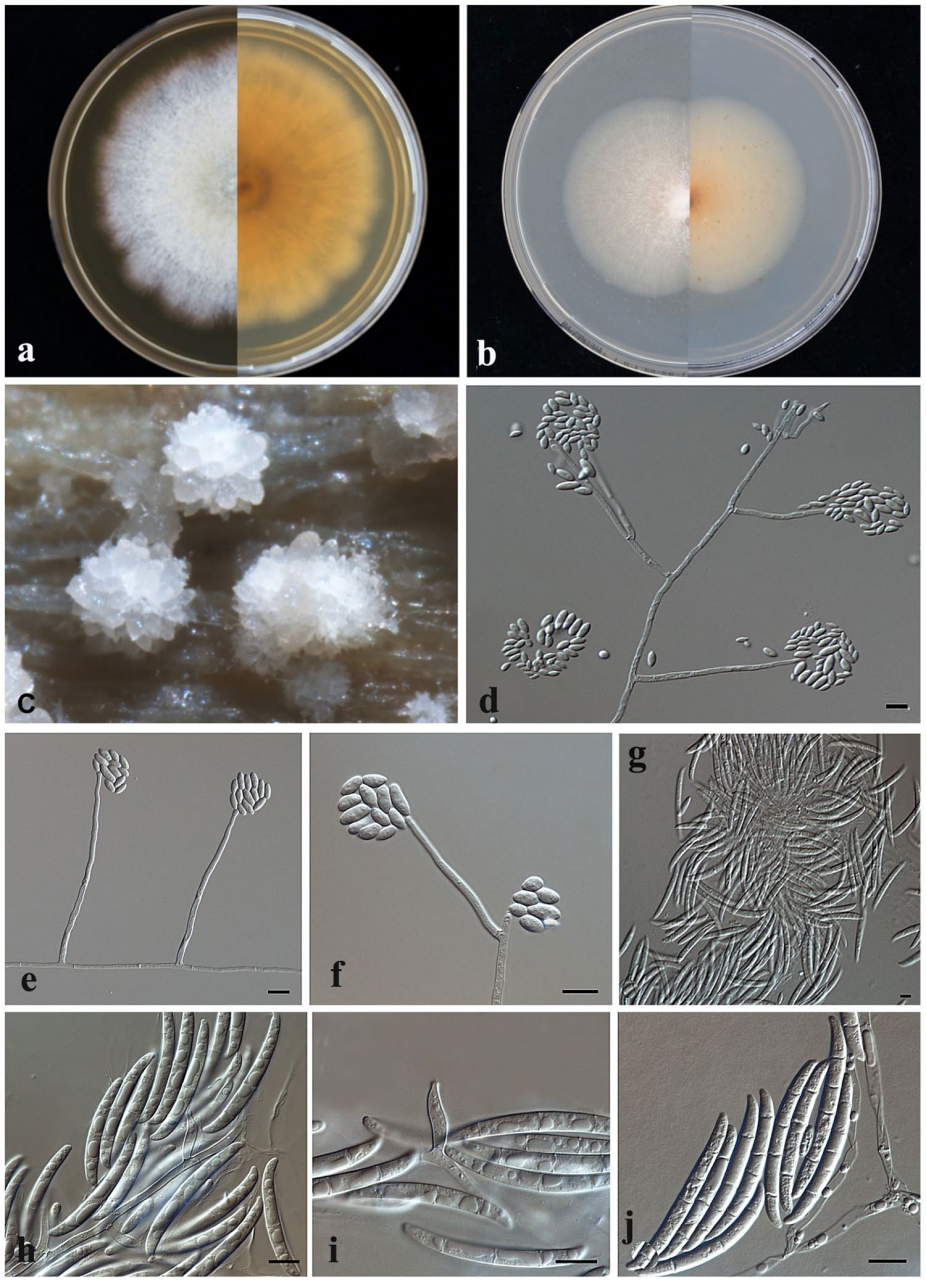
Morphological description of *Fusarium pisi* comb. nov. CBS 142372. (**a**) Growth on MEA (**b**) growth on PDA; (**c**) Sporodochia after 7 days appearing as white flowers on pieces of carnation leaf placed on CLA; (**d**–**f**) Long monophialides with false head and microconidia; (**f**) Minute conidia formed on short aerial conidiophores arising from hyphae; (**g**–**j**) Macroconidia abundant. Scale bar = 10 µm.

Following descriptions were based on CBS 142372 strain (Fs21 current study) growing in the darkness at 27 °C after 7 days on PDA, MEA and CLA. Colonies grew rapidly to a final diameter of 45 mm. Observed aerial mycelium cottony, white on MEA and white to milky on PDA (Fig. 4a,b). Reverse pigmentation yellowish to orange-brown. Sporodochia emerged after 7 days of incubation as white flowers on pieces of carnation leaf placed on CLA (Fig. 4c). Abundant production of erect, mononematous conidiophores from the agar surface and the aerial mycelium was also observed. Mononematous conidiophores were acremonium like and unbranched or occasionally branched once (Fig. 4d–f). Long conidiophores ranged from 2.1 to 162.2 µm with a mean length of 45.1 µm and 4.8 µm width. Short conidiophores ranged from 3.0 to 35.2 µm with mean length of 11.2 µm and 4.0 µm width at the base, terminating into a single sub-cylindrical phialide. Tip of the phialide with inconspicuous preclinical thickening, collarette not flared (4d-4f). Macroconidia was abundant, 4.3–46.1 × 5.4–6.2 μm, 2‒4 septate, slightly curved or arcuate with a rounded apical cell and wedge-shaped, weakly pedicellate basal cell (Fig. 4g–j). Microconidia ovoidal or with a rounded apex and truncate base, 2.5–12.7 × 1.5–2.1 μm (Fig. 4d–f). Chlamydospores absent.

Cardinal growth temperature test showed that the strain evaluated in this study had optimal development at 25–30 °C attaining diameter of 17 and 21 mm on PDA and SNA, respectively. Colonies showed no visible growth at 5 °C and at 35 and 40 °C on PDA. On SNA strain was still able to grow at 5 and 35 °C but not at 40 °C.

### Aggressiveness of selected FSSC isolates to pea in greenhouse assay

The severity of pea root rot averaged over isolates varied significantly among different species within the FSSC. *Fusarium pisi* (mean Disease Index, DI = 60.91) together with *F*. *redolens* (DI = 63.41) caused the highest overall disease severity, followed by *Fusisporium solani* (DI = 50.23) and the *F*. *solani* group (DI = 39.65) (Tukey HSD, *P* < 0.05). There were no significant differences in mean fresh or dry plant biomass of inoculated treatments and un-inoculated controls (data not shown).

**Figure 5:**
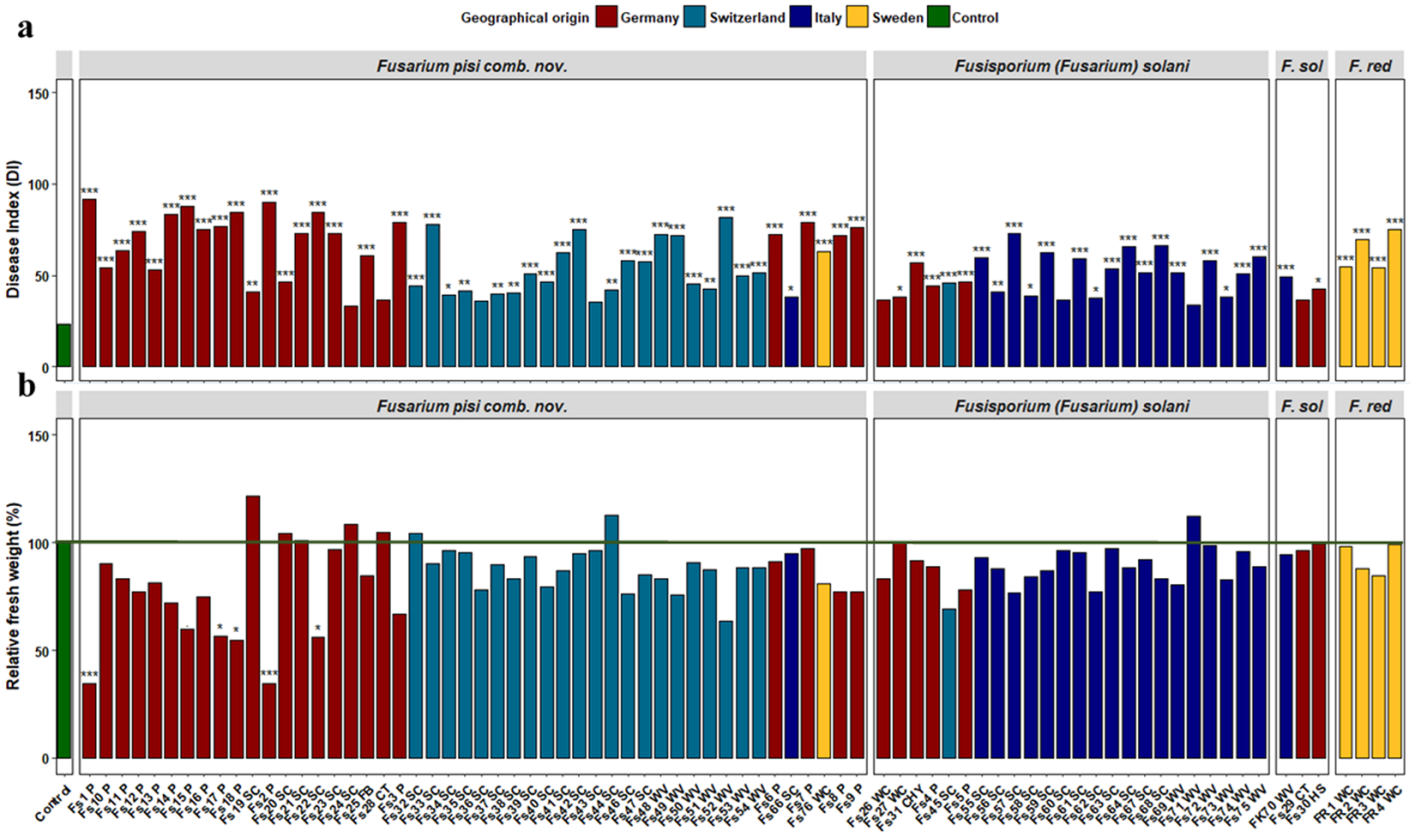
Effects of *Fusarium pisi* comb. nov., *Fusisporium* (*Fusarium*) *solani*, *F*. *solani* (F. sol) and *F*. *redolens* (F. red) isolates on root rot disease severity (**a**) and fresh plant biomass (**b**) of pea. The isolates that formed distinct groups based on the phylogenetic analysis and showed no strong phylogenetic relationship to any of previously defined species within the FSSC along with one isolate of *F*. *keratoplasticum* (FK70) are included in the *F*. *solani* group. Effects on the fresh plant biomass of inoculated plants are given relative to the un-inoculated control treatment performance which was set at 100% (green line). The letters in the suffix of each isolate ID number represent the host plant from which isolates were collected, where P = pea, SC = subterranean clover, WC = white clover, WV = winter vetch, FB = faba bean, HS = hibiscus, CHY = cherry, CT = compost. Asterisks above to the bars indicate significant difference from the un-inoculated control plants according to Dunnett’s t test. Symbols ***, **, and * indicate significance levels of P < 0.001, <0.01, and <0.05, respectively. Data presented are means of four replicate pots.

Significant variation in aggressiveness was also observed among individual isolates of *F*. *pisi, Fusisporium solani*, and those included in the *F*. *solani* group (Fig. 5). The majority of the isolates tested were pathogenic to pea. In general, weakly to moderately aggressive strains dominated the populations of the tested *Fusarium* species. Among the 48 *F*. *pisi* isolates, three isolates recovered from subterranean clover roots (Fs24, Fs36, Fs43) and one isolate collected from compost (Fs28) did not differ significantly in root rot severity from the un-inoculated control and were classified as non-aggressive (4/48, 8%). Among the pathogenic isolates, three (6%) were highly aggressive, 24 (50%) moderately aggressive, and 17 (35%) were weakly aggressive. Similar results were also observed for *Fusisporium solani* (Fig. 5a). Among the 24 isolates tested, three (Fs26, Fs60 and Fs71) recovered from the roots of white clover, subterranean clover and winter vetch, respectively, were classified as non-aggressive. The remaining isolates induced mild to modest symptoms on pea roots and were differentiated into weakly (12/24, 50%) and moderately aggressive (9/24, 38%). The two *F*. *solani* isolates collected from compost (Fs29) and hibiscus (Fs30), placed into one separate clade (lineage 1, Fig. 1) were rated as non- and weakly aggressive, respectively. The *F*. *keratoplasticum* isolate recovered from winter vetch in Italy (FK70), sorted in the *F*. *solani* group in Fig. 5, was weakly aggressive. Among the *F*. *redolens* isolates, two were weakly and two were moderately aggressive. Aggressiveness was not related to isolate phylogenetic position or the host plant from which it was recovered (Figs 1–5a).

In contrast to root rot severity, the effect of the tested isolates on pea biomass was much less pronounced. Significant reductions in fresh biomass compared to the corresponding control were observed only in plants inoculated with *F*. *pisi* isolates Fs1, Fs2, Fs17 and Fs18 recovered from pea roots, as well as for isolate Fs22 recovered from subterranean clover roots (Fig. 5b). In comparison to the un-inoculated control, dry plant biomass was significantly reduced only by isolate Fs1 (data not shown).

### Host range of *F*. *pisi* in greenhouse assays

*Fusarium pisi* was re-isolated from the surface sterilized roots in 58 out of the 62 accessions tested. Among the 58 successfully colonized accessions, 33 (including the three *Pisum* cultivars) were symptomatic and 25 accessions were asymptomatic hosts of *F*. *pisi* (Table 1). The fungus was not re-isolated from one *T*. *subterraneum* (acc. 1001) and three of the *T*. *repens* accessions (acc. 1965, 1968 and 2010). As assessed by plating surface sterilized root segments, these accessions were considered as non-hosts for the tested *F*. *pisi* isolate. In addition, several potentially pathogenic fungal species were isolated from the un-inoculated roots in 13 of the tested accessions: one *Pisum* cultivar, seven *Lathyrus*, four *T*. *subterraneum* and one *T*. *repens* accessions. Contaminants were usually *F*. *avenaceum* and *F*. *oxysporum*, several other *Fusarium* spp. and in one case *Didymella pinodella* (Table 1).

**Table 1:** Plant species and accessions tested for susceptibility to *F*. *pisi*, and the symptomatic (S), asymptomatic (AS) or non-host (NH) classification based on their response to infections measured 35 days after inoculation under controlled conditions.

Legume host	Common name	Code	DSR^1^ control	DSR inoculated	FW^2^ control (g)	FW^3^ change g and (%)	DW^2^ control (g)	DW^3^ change g and (%)	Host plant^4^
**PEA**									
*Pisum sativum* L. ssp. *sativum* convar. *speciosum*	Field pea	IPR83	3.0 FA	5.6**	3.91	−0.53* (−13.5)	0.48	−0.07 (−13.7)	S
*P*. *sativum* L. ssp. *sativum* convar. *speciosum*	Field pea	EFB 33	0.3	4.1***	4.71	−0.22 (−4.8)	0.63	+0.03 (4.8)	S
*P*. *sativum* L. ssp. *sativum* convar. *sativum*	Field pea	Santana	1.4	5.6***	3.21	+0.09 (2.8)	0.37	+0.05 (13.6)	S
**VETCHLINGS**									
*Lathyrus aphaca*	Yellow vetchling	L045	0.4 FA	1.8	1.00	−0.07 (−6.8)	0.14	−0.01 (−7.9)	AS
*L*. *dymenum*	Spanish vetchling	L1662	0.2 FA+PP	3.4**	2.09	−0.13 (−6.2)	0.26	−0.03 (−9.8)	S
*L*. *dymenum*	Spanish vetchling	L1660	0.8 FC	3.4**	2.59	+0.06 (2.1)	0.31	+0.01 (3.9)	S
*L*. *gorgoni*	Orange vetchling	L1663	0.6	2.2**	1.23	−0.31 (−25.4)	0.15	−0.04 (−27.4)	S
*L*. *inconspicuus*	Inconspicuous vetchling	L1672	1.2 FG	3.3*	0.95	−0.38* (−40.6)	0.12	−0.05* (−42.9)	S
*L*. *ochrus*	Winged vetchling	L1683	3.0 FA	4.4	3.05	+0.37 (12.2)	0.40	>+0.01 (0.1)	AS
*L*. *ochrus*	Winged vetchling	L1684	1.4 FA	3.6**	4.67	−0.82 (−17.5)	0.52	−0.11 (−21.0)	S
*L*. *sativus*	Chickling vetch	L1668	2.4 FT	5.2*	1.95	−0.14 (−7.4)	0.24	−0.06 (−23.6)	S
*L*. *sylvestris*	Flat vetchling	L1695	1.2	3.4*	2.99	−0.03 (−1.0)	0.35	−0.01 (−2.1)	S
**MEDICS**									
*Medicago arabica*	Spotted medick	1735	0.0	0.4	2.23	−0.07 (−3.2)	0.40	+0.02 (4.7)	AS
*M*. *arabica*	Spotted medick	211	0.1	1.3	1.78	−0.07 (−4.2)	0.27	+0.03 (9.8)	AS
*M*. *arabica*	Spotted medick	624	0.0	3.4*	2.34	−0.11 (−4.6)	0.37	+0.04 (11.8)	S
*M*. *orbicularis*	Button medick	44	0.0	2.8***	0.74	+0.32 (43.4)	0.11	+0.08** (65.7)	S
*M*. *orbicularis*	Button medick	46	0.1	2.3**	0.99	+0.02 (2.2)	0.15	+0.04 (28.3)	S
*M*. *polymorpha*	Burr medic	365	0.0	1.6	1.81	−0.10 (−5.2)	0.28	−0.04 (−13.6)	AS
**CLOVERS**									
*Trifolium angustifolium*	Narrowleaf crimson clover	20	0.2	1.6	0.68	+0.08 (12.2)	0.09	+0.01* (14.8)	AS
*T*. *arvense*	Haresfoot clover	1928	0.0	0.1	0.26	+0.22* (82.1)	0.06	+0.05** (89.9)	AS
*T*. *campestre*	Hop trefoil	1	0.0	0.2	0.53	+0.04 (7.6)	0.07	+0.02 (29.8)	AS
*T*. *diffusum*	Diffuse clover	906	0.2	3.2**	1.09	−0.52** (−47.8)	0.12	−0.05** (−43.5)	S
*T*. *palaestinum*	Palestine clover	910	0.2	4.0*	0.60	−0.13 (−21.4)	0.14	−0.05** (−37.5)	S
*T*. *repens*	White clover	1935	0.0	1.0	0.68	−0.03 (−4.3)	0.06	>+0.01 (0.2)	AS
*T*. *repens*	White clover	1936	0.0	0.5	1.04	−0.18 (−17.1)	0.13	−0.04 (−34.6)	AS
*T*. *repens*	White clover	1937	0.5	1.3	0.85	−0.07 (−8.2)	0.11	0.0 (0.0)	AS
*T*. *repens*	White clover	1954	0.0	0.4	0.77	−0.01 (−1.1)	0.09	−0.01 (−7.5)	NH
*T*. *repens*	White clover	1959	0.0 FO	0.6	0.86	+0.14 (16.3)	0.12	+0.02 (15.0)	AS
*T*. *repens*	White clover	1960	0.2	1.0	0.55	−0.01 (−1.6)	0.07	>−0.01 (−0.6)	AS
*T*. *repens*	White clover	1965	0.0	0.2	0.52	−0.12 (−23.5)	0.08	−0.02 (−23.5)	NH
*T*. *repens*	White clover	1968	0.0	0.4	0.54	+0.01 (2.0)	0.08	>−0.01 (−1.1)	NH
*T*. *repens*	White clover	1976	0.0	1.0	1.06	−0.24 (−22.3)	0.11	−0.02 (−16.2)	AS
*T*. *repens*	White clover	1977	0.0	0.5	0.76	−0.13 (−17.6)	0.09	−0.02 (−28.6)	AS
*T*. *repens*	White clover	2001	0.0	0.8	0.66	−0.20 (−29.6)	0.08	−0.01 (−8.6)	AS
*T*. *repens*	White clover	2010	0.0	1.0	0.72	+0.64* (88.5)	0.08	+0.05 (67.1)	AS
*T*. *subterraneum*	Subterranean clover	1001	1.4 FO	1.8	2.20	−0.21 (−9.4)	0.36	−0.03 (−7.6)	NH
*T*. *subterraneum*	Subterranean clover	1021	2.0 FO+FA	3.0	1.32	−0.15 (−11.0)	0.17	−0.03 (−18.3)	AS
*T*. *subterraneum*	Subterranean clover	1040	0.0	3.3**	1.01	−0.38 (−37.3)	0.12	−0.05 (−39.4)	S
*T*. *subterraneum*	Subterranean clover	1042	1.6 FO	3.2**	1.25	−0.15 (−11.6)	0.18	−0.03 (−16.4)	S
*T*. *subterraneum*	Subterranean clover	1065	3.6 FO	3.4	1.91	−0.14 (−7.3)	0.26	−0.02 (−6.6)	AS
*T*. *subterraneum*	Subterranean clover	1067	0.0	3.4**	1.09	−0.35 (−32.1)	0.13	−0.03 (−26.9)	S
*T*. *subterraneum*	Subterranean clover	1068	0.0	4.3**	0.93	−0.53 (−56.8)	0.10	−0.06 (−56.7)	S
*T*. *subterraneum*	Subterranean clover	Campeda	0.5	3.6**	0.54	+0.02 (3.2)	0.08	−0.01 (−8.2)	S
**VETCH**									
*Vicia articulata*	Bard vetch	924	0.4	2.2**	2.90	−0.32* (−11.0)	0.43	−0.10** (−24.0)	S
*V*. *benghalensis*	Purple vetch	1517	0.4	1.8	2.09	+0.20 (9.8)	0.31	+0.11** (34.2)	AS
*V*. *ervilia*	Bitter vetch	1527	0.0	3.4**	1.50	+0.06 (4.0)	0.19	+0.02 (10.8)	S
*V*. *fulgens*	Scarlet vetch	1532	0.0	1.0	1.85	−0.41 (−22.3)	0.39	−0.13* (−32.5)	S
*V*. *hirsuta*	Tiny vetch	1536	0.0	0.6	0.84	+0.08 (9.2)	0.12	+0.02 (14.5)	AS
*V*. *sativa*	Common vetch	1576	0.8	3.6**	2.96	−0.51** (−17.4)	0.40	−0.02 (−5.0)	S
*V*. *sativa*	Common vetch	1577	1.2	3.6**	2.34	+0.34 (14.5)	0.37	+0.02 (6.0)	S
*V*. *sativa*	Common vetch	1579	0.6	3.0**	2.03	+0.75** (37.1)	0.28	+0.13** (44.7)	S
*V*. *sativa*	Common vetch	1581	1.0	3.2**	2.82	+0.09 (3.1)	0.43	+0.03 (7.0)	S
*V*. *sativa*	Common vetch	1590	0.2	3.0**	2.82	−0.82** (−29.0)	0.44	−0.09* (−19.9)	S
*V*. *villosa*	Common vetch	1641	0.0	0.6	2.98	−0.30 (−10.2)	0.47	−0.07 (−14.0)	AS
*V*. *villosa*	Winter vetch	1642	0.0	0.2	2.22	+0.35* (15.6)	0.29	+0.17** (58.2)	AS
*V*. *villosa*	Winter vetch	1643	1.8	1.3	1.95	+0.71* (36.6)	0.33	+0.06 (18.2)	AS
*V*.*villosa* subsp. *varia*	Winter vetch	1644	0.2	1.4	2.99	−1.30** (−43.4)	0.46	−0.19** (−42.4)	S
**MELILOT**									
*Melilotus albus*	White melilot	1933	0.1	4.8***	0.82	−0.14 (−17.5)	0.08	+0.01 (16.6)	S
**BIRDS FOOTTREFOIL**									
*Lotus pedunculatus*	Marsh birds foot trefoil	1927	0.0	0.0	1.15	−0.10 (−9.1)	0.15	+0.01 (7.6)	AS
**RATTLEPOD**									
*Crotalaria ochroleuca*	Slender leaf rattlebox	n/a	0.6	4.0**	1.54	−0.56 (−36.7)	0.17	−0.07 (−37.3)	S
**GOATS RUE**									
*Galega officinalis*	Goats rue	162	0.2	1.4	0.88	+0.09 (10.5)	0.12	+0.01 (10.2)	S
***SCORPIONS TAIL***									
*Scorpiurus muricatus*	Prickly scorpions tail	69	0.8	5.4**	0.59	−0.30** (−50.9)	0.04	−0.02* (−39.1)	S
**FENUGREEK**									
*Trigonella foenum*−*graecum*	Fenugreek	409	2.0	7.8**	0.92	−0.72* (−78.3)	0.09	−0.07* (−73.2)	S

^1^DSR = disease severity rating of un−inoculated control plants, and additionally re-isolated fungi from surface sterilized roots, where FA = *Fusarium avenaceum*, FO = *F*. *oxysporum*, FC = *F*. *culmorum*, FG = *F*. *graminearum*, FT = *F*. *tricinctum* and PP = *Peyronellaea pinodella*;

^2^FW/DW = fresh/dry plant biomass of un-inoculated plants; ^3^FW/DW change = expressed as gram change in the biomass of the inoculated plants compared to corresponding un-inoculated control, and in parenthesis the biomass of the inoculated plants was expressed as a percentage change of the biomass of corresponding un-inoculated control plants.

^4^Symptomatic (S), asymptomatic (AS) and non-host (NH) accessions; With exception of *Pisum sativum* accession IPR83 provided by Instituto Agronomico do Parana (IAPAR), Brasil, all of the accessions were provided by Technical University of Munich, Germany.

Data were pairwise (2 by 2) analyzed by comparing inoculated treatments and corresponding un-inoculated controls using Dunn’s test. The symbols ***, **, and * indicate significance levels of *P* < 0.001, <0.01, and <0.05, respectively.

Inoculation with *F*. *pisi* resulted in significantly different levels of root rot disease severity among the tested legumes (Table 1). The highest overall disease severity rating (DSR) was observed on *Trigonella foenum-graecum* (mean DSR = 7.8), followed by *Scorpiurus muricatus* (DSR = 5.4), *Pisum* cultivars (DSR = 5.1), *Melilotus albus* (DSR = 4.8) *Crotalaria ochroleuca* (DSR = 4.0) and *Lathyrus* accessions (DSR = 3.4). Inoculated *Vicia, Medicago, Trifolium*, and *Galega* accessions showed lower overall disease symptoms, with mean severity ratings of 2.1, 2.0, 1.6 and 1.4, respectively. Symptoms of fungal infection were not observed in *Lotus pedunculatus* (Table 1).

Responses of individual accessions within *Lathyrus, Medicago, Trifolium* and *Vicia* genera to inoculation with *F*. *pisi* varied greatly (Table 1). For *Lathyrus*, with the exception of *L*. *aphaca* (L045), all accessions developed disease severity rating >2. Mean root rot severity ranged between 2.2 for *L*. *gorgoni* acc. L1663 and 5.2 for *L*. *sativus* acc. L1668. Similar responses were observed for *Medicago accessions*. Inoculated *M*. *arabica* (acc. 1735 and 211) and *M*. *polymorpha* (acc. 365) did not develop significant disease symptoms (DSR < 2), whereas *M*. *arabica* acc. 624 and *M*. *orbicularis* acc. 44 and 46 had DSR of 3.4, 2.8 and 2.3, respectively.

Significant variation in response to *F*. *pisi* was also observed for *Trifolium* accessions. Out of the 25 accessions tested, only seven were found to be susceptible (DSR > 2), with five belonging to *T*. *subterraneum* (DSR between 3.2 and 4.3), one to *T*. *diffusum* (acc. 906, DSR = 3.2) and one to *T*. *palestinum* (acc. 910, DSR = 4.0). The remaining 18 accessions had no symptoms or developed low levels of disease (DSR < 2). These included all *T*. *repens* accessions (n = 12), two *T*. *subterraneum* and one of each *T*. *angustifolium, T*. *arvense* and *T*. *campestre*.

Among the 14 *Vicia* accessions, six were considered susceptible based on the disease severity symptoms whereas five did not develop disease symptoms higher than two. For susceptible accessions, the mean DSR ranged between 2.2 for *V*. *articulata* acc. 924 and 3.6 for *V*. *sativa* accessions 1576 and 1577. *Vicia sativa* acc. 1576 and *V*. *villosa* subsp. *varia* acc. 1644 were also classified as symptomatic hosts in the absence of visible disease symptoms due to a significant loss of biomass (Table 1).

Low variability in response to *F*. *pisi* was found among the tested *Pisum* cultivars. Winter pea cv. EFB 33 (DSR = 4.1) was generally less susceptible to root rot compared to spring pea cv. Santana and cv. IPR83 (both DSR = 5.6) (Table 1).

Compared to the corresponding controls, fresh or dry plant biomass of inoculated treatments was significantly reduced only in *Pisum sativum* cv. IPR83, *Lathyrus incospicuus* acc. L1672, *Trifolium diffusum* acc. 906, *T*. *palestinum* acc. 910, *Vicia articulata* acc. 924, *V*. *sativa* acc. 1576 and 1590, *V*. *villosa* subsp. *varia* acc. 1644, *Scorpiurus muricatus* acc. 69 and *Trigonella foenum-graecum* acc. 409. Additionally, in some accessions, inoculation with *F*. *pisi* even resulted in a significant biomass increase (Table 1).

## Discussion

In the current study, the single gene phylogeny inferred from *tef1* gene sequences that included 79 isolates, as well as the single *rpb2* and the concatenated *tef1 – rpb2* phylogenetic tree topologies inferred for a selected subset of 28 isolates placed the examined strains in four lineages within the FSSC clade 3^17^. Most isolates were associated with two major lineages, the *F*. *pisi* (*F*. *solani* f. sp. *pisi*) lineage accommodating mainly German and Swiss isolates, and the *Fusisporium* (*Fusarium*) *solani s*. *str*. lineage accommodating mainly Italian isolates. The aggressiveness tests on pea that included a subset of 75 isolates confirmed the pathogenicity of most of the FSSC isolates tested. Aggressiveness was not related to isolate phylogenetic position or the host plant from which it was recovered.

The predominant share of the identified strains examined here (n = 51) belonged to *F*. *pisi*, suggesting a significant pathogenic potential of this species and/or a common prevalence in different host plants. Previous studies have established *F*. *pisi* as a primary causal agent of pea root rot and one of the main reasons for the decline of pea production worldwide^21,22^. While the result of the aggressiveness tests on pea in our study showed that the most aggressive isolates belonged to *F*. *pisi*, it is important to note that the tested population was dominated by weakly and moderately aggressive strains. In addition, non-pathogenic strains were also present in the population of the species. More importantly, our study showed that both, pathogenic and non-pathogenic isolates of *F*. *pisi* can be found in a variety of habitats under diverse agro-ecological conditions, and that the fungus is, in addition to pea, able to colonize roots of various hosts such as subterranean clover, white clover, winter vetch and faba bean under field conditions.

The ability of *F*. *pisi* to occupy diverse ecological niches and the significant variation in the aggressiveness of individual isolates observed in this study is consistent with the previous work of VanEtten^23^, who found large variation in pea plant symptom severity following inoculation with 152 *F*. *pisi* isolates collected from diverse habitats and geographical locations. While some isolates were highly aggressive to pea, the authors also reported the presence of non-pathogenic strains. Additional studies on the aggressiveness factors of *F*. *pisi* revealed that a number of enzymes released from the fungi have a major influence on their ability to cause disease. The capacity of the pathogen to degrade the phytoalexin pisatin by the activity of pisatin demethylase is one of the main determinants of its aggressiveness in pea. All naturally occurring isolates without this ability were essentially non-pathogenic^24^.

Out of the 26 additionally identified isolates in our study, 25 belonged to *Fusisporum solani* (*Fusarium solani* sensu stricto ‘5’), the species mainly considered as a causal agent of dry rot in potatoes and known as an opportunistic human pathogen^2^. However, the fungus has not been reported as part of the root rot complex of pea. As for *F*. *pisi*, our results suggest a lack of host specificity and the ability of *Fusisporium* (*Fusarium*) *solani* s. str. isolates to colonize various hosts under field conditions, as well as their potential to cause significant damage to pea. In addition, with few notable exceptions, the population of *F*. *pisi* isolates in our study mainly originated from German and Swiss environments, whereas the Italian isolates mainly comprised the *Fusisporium* (*Fusarium*) *solani s*. *str*. lineage. These results indicate a biogeographic distribution pattern of the FSSC species distribution with respect to the host plants.

One additionally identified isolate belonged to *F*. *keratoplasticum*, the species mainly associated with human eye infections^25^. Our data suggest considerable ecological plasticity of the fungus which possesses pathogenic potential on pea, and points to soil and plant debris as potential environmental sources of human infections. Included in *F*. *solani* group, the isolates Fs29 and Fs30 which were recovered from compost and hibiscus, respectively, represent at least one new phylogenetic lineage in the complex. Additional studies are needed to fully understand their ecological importance.

The greenhouse data on the host range of *F*. *pisi* further supports our observations that the species is not adapted to a particular host. Data from the single isolate inoculation indicate that the host range of *F*. *pisi* should be expanded to include 33 symptomatic and 25 asymptomatic hosts. Only one accession of subterranean clover and three accessions of white clover tested could be classified as non-hosts for *F*. *pisi* isolate Fs21 pointing to potential sources of resistance. A broader host range has been demonstrated for other special forms of *F*. *solani* previously. Studies on *F*. *solani* f. sp. *eumartii*, named by its specific pathogenicity to potato, revealed that the species was also pathogenic to pepper, eggplant and tomato^15^. Besides the *Solanaceae* family, the pathogen also infected maple (*Acer* sp.) and citrus (*Citrus* sp.) trees^26^. Similarly, *F*. *phaseoli* comb. nov^27^ (formerly *F*. *solani* f. sp. *phaseoli*) generally considered as a root rot pathogen of bean (*Phaseolus vulgaris*), has been associated with at least four other legume host plants^28^. In an extensive survey of *F*. *solani* f. sp. *glycines* associated with sudden death syndrome of soybean, Aoki *et al*.^27^ found that the disease is in fact caused by two phylogenetically and morphologically distinct species, *F*. *virguliforme* (formerly *F*. *solani* f. sp. *glycines*) and *F*. *tucumaniae* in North and South America, respectively. More recent studies conducted by Kolander *et al*.^14^ showed that *F*. *virguliforme* can cause disease in a range of legumes and non-legumes including alfalfa, red clover, white clover, pea, bean, sugar beet and canola. In addition, the authors demonstrated the ability of *F*. *virguliforme* to asymptomatically infect wheat, maize, ryegrass and lambsquarters, which are commonly grown in rotations with legumes. More recently, two new special forms have been assigned to the FSSC, namely *F*. *solani* ff. sp. *passiflorae*^7^ and *phalaenopsis*^8^. However, the authors did not provide sufficient molecular data that would allow comparison of their strains with already existing species within the FSSC, and based their results solely on the pathogenicity to the specific host evaluated on a narrow range of closely related plant species. Thus, whether such problems exist in these and other special forms within the complex remains to be investigated.

In addition to causing economically important crop diseases, *Fusarium* spp. in general and the members of the FSSC in particular that were previously considered solely as plant pathogens are being increasingly reported as causal agents of superficial and systemic infections in humans and animals^5,29^. For example, these so called trans-kingdom fungal pathogens have been demonstrated for *F*. *oxysporum* f. sp. *lycopersici*, *Fusisporium solani* s. str., *F*. *keratoplasticum* and *F*. *falciforme*^2,25,30,31^. Similarly, *F*. *pisi* has recently come into focus as a species of clinical relevance associated with human eye infection in the Netherlands (Al-Hatmi, unpublished data). This confirms that the species is adapted to many different habitats and supports the idea that *Fusarium* spp. can serve as a model for studying trans-kingdom pathogenicity in fungi^30^.

Our results provide new insights into the diversity of the FSSC associated with the legume host plants in Europe and provide another example for a wide host range of a single lineage within the complex. Currently the concept of forma specialis represents an informal rank in taxonomic classification^3,32^, and may deserve revision and formal taxonomic treatment.

## Material and Methods

### Isolates

A total of 79 FSSC isolates were collected for this study (Table 2). Among these, 18 isolates were recovered from pea (*Pisum sativum*), 39 from subterranean clover (*Trifolium subterraneum*), 3 from white clover (*T*. *repens*) and 14 from winter vetch (*Vicia villosa*). The isolates originated from Germany (n = 28), Switzerland (n = 24), Italy (n = 21) and Sweden (n = 1). Additionally, we included one isolate from faba bean (*V*. *faba*), and expanded the isolates associated with legumes with two isolates collected from compost and two (one of each) recovered from an infected hibiscus (*Hibiscus* sp.) and cherry tree (*Prunus* sp.), all from Germany (Table 2). All isolates were collected in the period between 2009 and 2016, morphologically identified as *F*. *solani* and maintained as single-spore cultures at the Internal Culture Collection of the Ecological Plant Protection Department at University of Kassel. Four isolates of *F*. *redolens*, recovered from white clover grown in Sweden, were additionally included in this study.

**Table 2:** *Fusarium isolates* subjected to phylogenetic analysis and evaluated for aggressiveness to pea in greenhouse experiment 1.

Isolate ID^1^	Species^2^	Host/Substrate^3^	Geographical origin	Year	GenBank accession numbers^4^	
					*tef1*	*rpb2*
Fs1	*Fusarium pisi*	Pea (*Pisum sativum*)	Germany, Frankenhausen, Hessen	2013	KY556491	—
Fs2	*F*. *pisi*	Pea	Germany, Frankenhausen, Hessen	2013	KY556463	—
Fs3	*F*. *pisi*	Pea	Germany, Frankenhausen, Hessen	2013	KY556448	—
Fs4	*Fusisporium solani*	Pea	Germany, Frankenhausen, Hessen	2013	KY556500	KY556544
Fs5	*Fusisporium solani*	Pea	Germany, Frankenhausen, Hessen	2013	KY556511	—
Fs6	*F*. *pisi*	Pea	Germany, Neu Eichenberg, Hessen	2013	KY556459	—
Fs7	*F*. *pisi*	Pea	Germany, Neu Eichenberg, Hessen	2013	KY556466	—
Fs8	*F*. *pisi*	Pea	Germany, Neu Eichenberg, Hessen	2013	KY556450	—
Fs9	*F*. *pisi*	Pea	Germany, Frankenhausen, Hessen	2013	KY556451	—
Fs10	*F*. *pisi*	Pea	Germany, n/a	2009	KY556452	—
Fs11	*F*. *pisi*	Pea	Germany, Frankenhausen, Hessen	2013	KY556497	—
Fs12	*F*. *pisi*	Pea	Germany, Frankenhausen, Hessen	2013	KY556447	—
Fs13	*F*. *pisi*	Pea	Germany, Neu Eichenberg, Hessen	2013	KY556453	—
Fs14	*F*. *pisi*	Pea	Germany, Frankenhausen, Hessen	2013	KY556449	—
Fs15	*F*. *pisi*	Pea seed	Germany, Neu Eichenberg, Hessen	2012	KY556492	—
Fs16	*F*. *pisi*	Pea seed	Germany, Neu Eichenberg, Hessen	2011	KY556493	—
Fs17	*F*. *pisi*	Pea seed	Germany, Neu Eichenberg, Hessen	2011	KY556471	—
Fs18	*F*. *pisi*	Pea seed	Germany, Neu Eichenberg, Hessen	2011	KY556458	KY556526
Fs19	*F*. *pisi*	Subterranean clover (*Trifolium subterranean*)	Germany, Neu Eichenberg, Hessen	2013	KY556472	KY556535
Fs20	*F*. *pisi*	Subterranean clover	Germany, Freising-Weihenstephan, Bavaria	2015	KY556488	KY556536
Fs21	*F*. *pisi*	Subterranean clover	Germany, Neu Eichenberg, Hessen	2013	KY556454	KY556537
Fs22	*F*. *pisi*	Subterranean clover	Germany, Neu Eichenberg, Hessen	2014	KY556473	KY556527
Fs23	*F*. *pisi*	Subterranean clover	Germany, Neu Eichenberg, Hessen	2013	KY556455	KY556538
Fs24	*F*. *pisi*	Subterranean clover	Germany, Neu Eichenberg, Hessen	2013	KY556474	—
Fs25	*F*. *pisi*	Faba bean (*Vicia faba*)	Germany, Freising, Bavaria	2015	KY556460	—
Fs26	*Fusisporium solani*	White clover (*Trifolium repens)*	Germany, Neu Eichenberg, Hessen	2014	KY556517	KY556542
Fs27	*Fusisporium solani*	White clover	Germany, Neu Eichenberg, Hessen	2014	KY556501	—
Fs28	*F*. *pisi*	Compost	Germany, Hannover, Lower Saxony	2014	KY556475	KY556528
Fs29	*F*. *solani*	Compost	Germany, Hannover, Lower Saxony	2014	KY556524	KY556552
Fs30	*F*. *solani*	Hibiscus dying branch (*Hibiscus* sp.)	Germany, Witzenhausen, Hessen	2015	KY556525	KY556553
Fs31	*Fusisporium solani*	Cherry dying branch (*Prunus* sp.)	Germany, Witzenhausen, Hessen	2016	KY556520	KY556549
Fs32	*F*. *pisi*	Subterranean clover	Switzerland, Reckenholz, Canton Zurich	2013	KY556476	—
Fs33	*F*. *pisi*	Subterranean clover	Switzerland, Reckenholz, Canton Zurich	2013	KY556486	KY556529
Fs34	*F*. *pisi*	Subterranean clover	Switzerland, Reckenholz, Canton Zurich	2013	KY556484	KY556530
Fs35	*F*. *pisi*	Subterranean clover	Switzerland, Reckenholz, Canton Zurich	2013	KY556482	KY556539
Fs36	*F*. *pisi*	Subterranean clover	Switzerland, Reckenholz, Canton Zurich	2013	KY556487	—
Fs37	*F*. *pisi*	Subterranean clover	Switzerland, Reckenholz, Canton Zurich	2013	KY556495	—
Fs38	*F*. *pisi*	Subterranean clover	Switzerland, Reckenholz, Canton Zurich	2015	KY556456	—
Fs39	*F*. *pisi*	Subterranean clover	Switzerland, Reckenholz, Canton Zurich	2013	KY556477	—
Fs40	*F*. *pisi*	Subterranean clover	Switzerland, Reckenholz, Canton Zurich	2013	KY556478	—
Fs41	*F*. *pisi*	Subterranean clover	Switzerland, Reckenholz, Canton Zurich	2013	KY556464	—
Fs42	*F*. *pisi*	Subterranean clover	Switzerland, Reckenholz, Canton Zurich	2013	KY556485	KY556531
Fs43	*F*. *pisi*	Subterranean clover	Switzerland, Reckenholz, Canton Zurich	2013	KY556479	KY556532
Fs44	*F*. *pisi*	Subterranean clover	Switzerland, Reckenholz, Canton Zurich	2013	KY556470	KY556533
Fs45	*Fusisporium solani*	Subterranean clover	Switzerland, Reckenholz, Canton Zurich	2013	KY556521	KY556543
Fs46	*F*. *pisi*	Subterranean clover	Switzerland, Reckenholz, Canton Zurich	2015	KY556467	KY556534
Fs47	*F*. *pisi*	Subterranean clover	Switzerland, Reckenholz, Canton Zurich	2015	KY556461	—
Fs48	*F*. *pisi*	Winter vetch	Switzerland, Reckenholz, Canton Zurich	2015	KY556489	—
Fs49	*F*. *pisi*	Winter vetch	Switzerland, Reckenholz, Canton Zurich	2015	KY556490	KY556540
Fs50	*F*. *pisi*	Winter vetch	Switzerland, Reckenholz, Canton Zurich	2015	KY556480	—
Fs51	*F*. *pisi*	Winter vetch	Switzerland, Reckenholz, Canton Zurich	2015	KY556468	—
Fs52	*F*. *pisi*	Winter vetch	Switzerland, Reckenholz, Canton Zurich	2015	KY556496	—
Fs53	*F*. *pisi*	Winter vetch	Switzerland, Reckenholz, Canton Zurich	2015	KY556465	—
Fs54	*F*. *pisi*	Winter vetch	Switzerland, Reckenholz, Canton Zurich	2015	KY556483	—
Fs55	*Fusisporium solani*	Subterranean clover	Italy, Localita’ Riello, Viterbo	2015	KY556515	—
Fs56	*Fusisporium solani*	Subterranean clover	Italy, Localita’ Riello, Viterbo	2015	KY556502	—
Fs57	*Fusisporium solani*	Subterranean clover	Italy, Localita’ Riello, Viterbo	2015	KY556503	KY556545
Fs58	*Fusisporium solani*	Subterranean clover	Italy, Localita’ Riello, Viterbo	2015	KY556498	KY556546
Fs59	*Fusisporium solani*	Subterranean clover	Italy, Localita’ Riello, Viterbo	2015	KY556504	—
Fs60	*Fusisporium solani*	Subterranean clover	Italy, Localita’ Riello, Viterbo	2015	KY556518	KY556547
Fs61	*Fusisporium solani*	Subterranean clover	Italy, Localita’ Riello, Viterbo	2015	KY556505	—
Fs62	*Fusisporium solani*	Subterranean clover	Italy, Localita’ Riello, Viterbo	2015	KY556509	—
Fs63	*Fusisporium solani*	Subterranean clover	Italy, Localita’ Riello, Viterbo	2013	KY556519	—
Fs64	*Fusisporium solani*	Subterranean clover	Italy, Localita’ Riello, Viterbo	2015	KY556510	—
Fs66	*F*. *pisi*	Subterranean clover	Italy, Localita’ Riello, Viterbo	2015	KY556469	—
Fs67	*Fusisporium solani*	Subterranean clover	Italy, Localita’ Riello, Viterbo	2015	KY556499	—
Fs68	*Fusisporium solani*	Subterranean clover	Italy, Localita’ Riello, Viterbo	2015	KY556506	—
Fs69	*Fusisporium solani*	Winter vetch	Italy, Localita’ Riello, Viterbo	2015	KY556522	KY556550
FK70	*F*. *keratoplasticum*	Winter vetch	Italy, San Piero a Grado, Tuscany	2014	KY556523	—
Fs71	*Fusisporium solani*	Winter vetch	Italy, Localita’ Riello, Viterbo	2015	KY556507	KY556548
Fs72	*Fusisporium solani*	Winter vetch	Italy, San Piero a Grado, Tuscany	2014	KY556512	KY556551
Fs73	*Fusisporium solani*	Winter vetch	Italy, San Piero a Grado, Tuscany	2015	KY556508	—
Fs74	*Fusisporium solani*	Winter vetch	Italy, San Piero a Grado, Tuscany	2015	KY556514	—
Fs75	*Fusisporium solani*	Winter vetch	Italy, San Piero a Grado, Tuscany	2015	KY556513	—
Fs76	*F*. *pisi*	White clover	Sweden, n/a, Upsala	2014	KY556462	—
Fs77	*F*. *pisi*	Subterranean clover	Germany, Neu Eichenberg, Hessen	2014	KY556457	KY556541
Fs78	*F*. *pisi*	Subterranean clover	Switzerland, Reckenholz, Canton Zurich	2013	KY556494	—
Fs79	*Fusisporium solani*	Subterranean clover	Italy, Localita’ Riello, Viterbo	2015	KY556516	—
Fs80	*F*. *pisi*	Subterranean clover	Germany, Neu Eichenberg, Hessen	2014	KY556481	—
FR1	*F*. *redolens*	White clover	Sweden, n/a, Upsala	2014	KY556443	—
FR2	*F*. *redolens*	White clover	Sweden, n/a, Upsala	2015	KY556444	—
FR3	*F*. *redolens*	White clover	Sweden, n/a, Upsala	2014	KY556445	—
FR4	*F*. *redolens*	White clover	Sweden, n/a, Upsala	2014	KY556446	—

^1^All isolates with exception of Fs77, Fs78, Fs79 and Fs80 were tested for aggressiveness on pea in greenhouse experiment 1;

^2^Isolates that formed distinct groups based on the phylogenetic analysis and showed no strong phylogenetic relationship to any of the previously defined species within the FSSC were termed as *F*. *solani*;

^3^Unless indicated differently isolates were collected from infected root system;

^4^GenBank accession numbers for translation elongation factor 1-alpha (*tef1*) partial sequences and the second-largest subunit of RNA polymerase II (*rpb2*) gen region (selected isolates). n/a = not available.

### DNA extraction, PCR amplification and sequencing

Genomic DNA was extracted from cultures actively growing on half strength PDA agar plates (½ strength PDA; 19 g/l Difco PDA and 10 g/l agar) using the CTAB (cetyltrimethylammonium bromide) protocol described by Doyle and Doyle^33^. All DNA were diluted 20 times in milli-Q water and stored at −20 °C before use.

A portion of the translation-elongation factor 1 alpha (*tef1*) gene was amplified using primer pairs EF1 and EF2^34^. Based on the *tef1* tree topology, 28 strains were selected and the second-largest subunit of RNA polymerase II (*rpb2*) was amplified using primers RPB2-5F2^35^ and fRPB2-7cR^36^. Each polymerase chain reaction (PCR) had a total volume of 50 μl and contained 1 μl of diluted genomic DNA, 10× TrueStart Hot Start Taq Buffer [200 mM Tris-HCl (pH 8.3 at 25 °C), 200 mM KCl, 50 mM (NH_4_)_2_SO_4_], 2.5 mM MgCl_2_, 0.2 mM of each of the dNTP, 0.4 µM of each primer, and 1 unit TrueStart Hot Start *Taq* DNA Polymerase (ThermoFisher Scientific, Darmstadt, Germany). The PCR reactions were performed in a Biometra TAdvanced Thermal Cycler (Applied Biosystems, Foster City, California, USA). Conditions for amplification for the *tef1* gene region were an initial denaturation step of 3 min at 95 °C, followed by 30 cycles of denaturation (95 °C for 30 s), annealing (53 °C for 30 s) and elongation (72 °C for 45 s). The final elongation step was conducted at 72 °C for 7 min. For the *rpb2* loci amplification consisted of 5 cycles of 45 s at 94 °C, 45 s at 60 °C and 2 min at 72 °C, then 5 cycles with a 58 °C annealing temperature and 30 cycles with a 54 °C annealing temperature^37^.

Amplicons were purified using the DNA Clean & Concentrator kit (Zymo Research, Freiburg, Germany) according to the manufacturer’s instructions and sequenced in both directions either by Eurofins Genomics (Ebersberg, Germany) or by MacroGen (Amsterdam, Netherlands) with the above-mentioned primers.

### Phylogenetic analyses

Obtained row sequences were assembled and errors identified and corrected in MEGA v6^38^. Partial sequences of *tef1* and *rpb2* were used as queries for the Fusarium-ID v. 1.0 database^20^, and the *Fusarium* MLST databases (http://www.cbs.knaw.nl/fusarium ref.^39^) to confirm the taxonomic assignments of the isolates. The sequences were aligned with sequences of reference strains retrieved from the GenBank using MAFFT v.7 (http://mafft.cbrc.jp/alignment/server/index.html ref.^40^) and the alignments were adjusted manually with MEGA v6. The phylogenetic analyses, including the majority of known *Fusarium* species within the FSSC, was performed on *tef1* and *rpb2* sequences separately as well as on a combined data set (28 selected strains). The strains used in these analyses and GenBank accession numbers of the sequences are listed in Table 2 and Table 3. A bootstrapped Maximum-Likelihood (ML) analysis was performed using the RAxML-VI-HPC v. 7.0.3 with non-parametric bootstrapping and 1000 replicates implemented on the Cipres portal (http://www.phylo.org ref.^41^). For the outgroup purposes, the *F*. *redolens* and *F*. *thapsinum* (H05-557S-1 DCPA and CBS 130176) were used to generate the phylogenetic trees.

**Table 3:** Reference strains sourced from the NCBI GenBank database used to examine phylogenetic relationships among collected isolates.

Species	Strain number	GenBank accession numbers^1^	
		*tef1*	*rpb2*
*Fusarium solani*	CBS 119996	HE647962.1	n/a
*Fusarium petroliphilum*	CBS 135955	n/a	KJ867426
*Fusarium falciforme*	CBS 138963	KT716213.1	n/a
*Fusarium phaseoli*	CBS 265.50	HE647964.1	n/a
*Fusarium paranaense*	CML 1988	KF597819.1	n/a
*Fusarium solani* f. sp. *piperis*	CML 2190	JX657675.1	n/a
*Fusarium rectiphorum*	FRC S1831	DQ247509.1	n/a
*Fusarium haematococcum*	FRC S1832	DQ247510.1	n/a
*Fusarium kurunegalense*	FRC S1833	DQ247511.1	n/a
*Fusarium kelerajum*	FRC S1837	DQ247516.1	n/a
*Fusarium mahasenii*	FRC S1845	DQ247513.1	n/a
*Fusarium* cf. *ensiforme*	FRC S1847	JF433028.1	n/a
*Fusarium keratoplasticum*	FRC S2477	KR673939.1	KR673969
*Fusarium solani*	FRC S485	DQ247312.1	n/a
*Fusarium ambrosium*	NRRL 20438	AF178332.1	n/a
*Fusarium illudens*	NRRL 22090	AF178326.1	JX171601
*Fusarium*sp. *cucurbitae* MPI	NRRL 22098	n/a	EU329489
*Fusarium* sp.	NRRL 22101	n/a	EU329490.1
*Fusarium* sp. *cucurbitae* MPV	NRRL 22141	n/a	EU329491
*Fusarium solani* f. sp. *mori*	NRRL 22157	AF178359.1	EU329493
*Fusarium* sp. *robiniae*	NRRL 22161	n/a	EU329494
*Fusarium solani* f. sp. *xanthoxyli*	NRRL 22163	AF178328.1	n/a
*Neocosmospora vasinfecta*	NRRL 22166	AF178350.1	EU329497
*Fusarium* sp.	NRRL 22178	n/a	EU329498
*Fusarium* sp.	NRRL 22230	n/a	EU329499
*Fusarium phaseoli*	NRRL 22276	n/a	JX171608
*Fusarium* sp.	NRRL 22278	n/a	EU329501
*Fusarium ambrosium*	NRRL 22354	n/a	EU329504
*Fusarium kurunegalense*	NRRL 22387	n/a	EU329505
*Fusarium rectiphorus*	NRRL 22396	n/a	EU329508
*Fusarium* sp. *batatas*	NRRL 22400	n/a	EU329509
*Fusarium solani* f. *batatas*	NRRL 22402	AF178344.1	n/a
*Fusarium solani* (FSSC6)	NRRL 22404	DQ247594.1	n/a
*Fusarium* sp.	NRRL 22436	n/a	EU329511
*Fusarium*.sp. *piperus*	NRRL 22570	n/a	EU329513
*Fusarium sp*.	NRRL 22579	n/a	EU329515
*Fusarium* sp. (FSSC 13)	NRRL 22586	AF178353.1	n/a
*Fusarium plagianthi*	NRRL 22632	AF178354.1	n/a
*Fusarium sp*. *plagianthi*	NRRL 22632	n/a	EU329519
*Fusarium brasiliense*	NRRL 22678	JQ670133.1	n/a
*Fusarium tucumaniae*	NRRL 22744	DQ247651.1	n/a
*Fusarium solani* f. sp. *pisi*	NRRL 22820	AF178355.1	EU329532
*Fusarium virguliforme*	NRRL 22825	n/a	GU170599
*Fusarium solani*	NRRL 25083	JF740714.1	n/a
*Fusarium redolens*	NRRL 25123	JF740748.1	n/a
*Fusarium lichenicola*	NRRL 28030	DQ246877.1	n/a
*Fusarium brasiliense*	NRRL 31757	n/a	EU329565
*Fusarium* sp. (FSSC 12d)	NRRL 32309	DQ246937.1	n/a
*Fusarium lichenicola*	NRRL 34123	n/a	EU329635
*Fusarium virguliforme*	NRRL 36899	FJ919494.1	n/a
*Fusarium* sp.	NRRL 45880	n/a	EU329640
*Fusarium pseudensiforme*	NRRL 46517	KC691555.1	KC691674
*Fusarium solani* (FSS5)	NRRL 46643	GU250544	GU250729
*Fusarium euwallaceae*	NRRL 54722	JQ038007.1	n/a
*Fusarium petroliphilum*	NRRL 54988	KC808210.1	n/a
*Fusarium* sp.	44a GJS 09–1459^2^	KT313606.1	n/a
*Fusarium* cf. *solani*	B8659^2^	HM852045.1	n/a
*Fusarium solani* f. sp. *cucurbitae*	Fsm731^2^	KC711041.1	n/a
*Fusarium thapsinum*	H05-557S-1 DCPA^2^	JX268965.1	n/a
*Fusarium striatum*	SQHI003^2^	KP715415.1	n/a

CBS = Centraalbureau voor Schimmelcultures—Fungal Biodiversity Center, Utrecht, The Netherlands; CML = Coleção Micológica de Lavras, Departamento de Fitopatologia, Universidade Federal de Lavras, Lavras, Minas Gerais, Brazil; FRC = Specimen number in the *Fusarium* Research Center, Pennsylvania State University; NRRL Agricultural Research Service Culture Collection, Peoria, Illinois USA;

^1^Reference strains GenBank accession numbers for translation elongation factor 1-alpha (*tef1*) partial sequences and the second-largest subunit of RNA polymerase II (*rpb2*) gen region.

^2^Unknown culture collections; n/a = the sequences were either not available or not applicable to the current study.

### Growth rates and morphological characterization of *Fusarium pisi* (*F*. *solani* f. sp. *pisi*)

The isolate Fs21 (CBS 142372) collected from the roots of subterranean clover was chosen as the representative strain to study morphological characters. Cardinal growth rates were determined at eight temperatures (5–40 °C) at 5 °C intervals in darkness following methods adopted from Nalim *et al*.^31^. Briefly, a five mm diameter plugs were taken from the actively growing edge of a 15 days old colony cultured on potato dextrose agar (PDA; 39 g/l Difco), and placed mycelium side down one cm from the edge of a fresh PDA and synthetic nutrient-poor agar^42^ (SNA). Colony radius was measured after five days from the edge of the inoculum plug to the most distant part of the colony. Average growth rates were calculated from three replicate plates for each respective temperature and expressed as diametric growth per 24 h.

Plates for colony morphology and colors were prepared by placinga five mm agar plug in the center of PDA, malt extract agar (MEA, Oxoid, UK) and carnation leaf agar^4^ (CLA). Culture plates were grown at 27 °C in the dark and examined seven days after inoculation. For microscopic observations, a block of ca. one cm SNA agar was cut and placed on a microscopic slide, inoculated with the fungus and covered with a No. 1 cover glass. Slides were examined in a drop of lactic acid with cotton blue, and the pictures were taken with a Jenoptik ProgRes® digital camera (JENOPTIK, Germany) attached to a Zeiss Axiosskop2 plus microscope. A minimum of 20 measurements were made per structure using the CapturePro 2.8 (JENOPTIK, Germany) software.

### Greenhouse experiments

#### Aggressiveness of selected FSSC isolates to pea

To compare aggressiveness (relative ability of the pathogen/isolate to colonize and cause damage to plants^43^) and to determine whether the FSSC strains from non-pea hosts are capable of causing disease on pea, a total of 75 isolates were tested in a greenhouse assay. The aggressiveness test included 48 isolates of *F*. *pisi*, 24 isolates of *Fusisporium solani*, 2 isolates of *F*. *solani*, and 1 isolate of *F*. *keratoplasticum*. In this study, the isolates that formed distinct groups based on the phylogenetic analysis and showed no strong phylogenetic relationship to any of the previously defined species within the FSSC were included in the *F*. *solani* group. Four isolates of *F*. *redolens* were also included in this experiment. The geographic origin and the host plants from which the isolates were collected are given in Table 2.

To prepare inoculum, each *Fusarium* isolate was cultured on ½ strength PDA at room temperature under alternating cycles of 12 h blacklight blue (BLB) fluorescent light (F40; range 315–400 nm with the peak at 365 nm) and 12 h darkness. After 15 days, spores were washed with sterile distilled water and enumerated in the suspension with a Fuchs Rosenthal hemocytometer.

Seeds of field pea cv. Santana were surface sterilized in 70% ethanol for 5 five minutes and rinsed with distilled water prior to planting. Four pea seeds (germination rate of 98%) were then planted into 500 ml pots filled with autoclaved sand, and 2 × 10^4^ spores g^−1^ substrate of the respective isolate was applied to each pot. Un-inoculated controls were mock inoculated with sterile distilled water. Four replicate posts were sown per treatment and arranged in a completely randomized design. Experimental plants were kept in the greenhouse at 19 °C day and 16 °C night temperature. Natural day light was additionally supplemented with high-pressure sodium lamps (400 W) in order to provide a photoperiod of 16 h light day^−1^. Plants were watered daily with tap water and additionally fertilized with complex N:P:K fertilizer Wuxal Super (8:8:6 + microelements). A total of 120 mg of N l^−1^ of substrate was divided into four portions and given over the course of the experiment.

After 42 days of growing, plants were removed from pots, and the roots were separated from the shoots. Above ground plant parts of each pot were weighted and dried at 105 °C until constant weight was attained. Roots were washed under running tap water, and root rot severity was assessed using a visual 0–8 score scale based on external and internal root tissue discoloration levels adopted from Pflughöft^44^. The external disease severity was rated as follows: disease severity rating (DSR) 0 = no symptoms, 1 = streaks at the transition zone, epicotyl or hypocotyl, 2 = brown lesion cover up to 50% of root perimeter, 3 = brown-black lesion cover 51 to 99% of root perimeter, 4 = black lesion cover 100% of stem perimeter, 5 = black lesion spread up to 30–49% of the tap root, 6 = black lesions spread up to 50 to 70% of the tap root, 7 = black lesions spread >70% of the tap root, 8 = dead plant. The roots were then cut transversally across the lesions and internal disease severity was rated, where 0 = no visible symptoms, 1 = epidermis/rhizodermis is brown to black, 2 = brown discoloration of cortical tissues, 3 = cortical tissues is partially black, but the center and endodermis are still healthy, 4 = cortex tissue is completely black, 5 = cortex tissue begins to rot (bursting of epicotyl or rhizodermis on the root), 6 = cortex tissue is completely rotten, 7 = shedding of the cortex tissue and endodermis, and 8 = dead plant. Consequently, a disease severity index (DI) between 0 and 100 was calculated for each pot using equation (1):

1 $${\rm{DI}}=\sum \frac{({\rm{SR}}\,\times \,{\rm{NR}})}{{\rm{Nt}}\,\times \,{\rm{MR}}}\,\ast \,100\,$$

where, SR = Mean external and internal disease severity rating (DSR), NR = Number of infected plants having that DSR, Nt = Total number of plants assessed, MR = Maximum rating scale number.

Four distinct aggressiveness classes were then assigned relative to the un-inoculated control and based on the gradual increase of severity of symptoms following inoculation, where: DI = 0–36 – non-aggressive; DI = 37–55 – weakly aggressive; DI = 56–85 – moderately aggressive; and DI = 86–100 – highly aggressive. A threshold disease index of 36 for classifying an isolate as aggressive was chosen because factors other than inoculation caused low levels of root discoloration in un-inoculated control plants. Up to this level the DI of inoculated treatments was in the same range as the DI of the un-inoculated control; *e*.*g*. mean DI of un-inoculated controls was 24 (±12) while the mean DI of inoculated treatment was ≤36. In addition, up to this level there was no statistically significant difference in DI of inoculated treatments and un-inoculated control.

Twenty one different inoculation treatments were selected at random and the fungi were re-isolated from the surface sterilized roots (1% NaOCl, 3 roots per treatment) and identified morphologically to confirm recovery of the isolate.

#### Evaluation of host range of *Fusarium pisi*

To determine the host range and evaluate plant response to inoculation with *F*. *pisi*, 60 accessions of 10 legume genera were tested in a greenhouse assay. This study was conducted over a set of four consecutive experiments. In each experiment two field pea cultivars, cv. Santana and cv. EFB 33, were included as additional controls (Table 1).

The *F*. *pisi* isolate (Fs21) classified as moderately aggressive to pea in experiment 1 was selected for the inoculation experiments. The inoculum was prepared by incubating the strain for 10 days in aerated malt extract broth (MEB, 17 g/l) at 20 °C under constant agitation/shaking at 100 rpm. After 10 days of incubation, conidia were collected by filtration and enumerated in suspension as described above.

Preliminary studies on seed germination using untreated seeds showed that the majority of accessions chosen for this experiment had a very low germination rate. Thus, to ensure adequate seedling emergence, seeds of all plant accessions, with the exception of pea, were treated with 97% sulfuric acid for 4 min, rinsed in distilled water and germinated for 48 h on wet filter paper in Petri dishes at room temperature. Pea seeds were treated with 70% ethanol prior to placing on wet filter paper. Single pre-germinated seeds were then transplanted into 200 ml pots filled with autoclaved sand. Each treatment consisted of five replicates with one germinated seed sown per pot. The experiment was arranged in a completely randomized design and the pots were inoculated 24 h after transplanting with 2 × 10^4^ spores g^−1^ substrate. Plants were kept in the greenhouse for five weeks under the conditions described for experiment 1. After five weeks of growing, plants were harvested and the biomass and disease severity (external root tissue discoloration levels only) were assessed as described above.

Cultural methods in combination with disease severity data were used to determine the host range of *F*. *pisi* on tested plants. Three randomly selected roots from each treatment were surface-sterilized in 0.5% NaOCl for 10 s, thoroughly washed in distilled water and placed on filter paper under a laminar flow hood for 1 h to dry. Subsequently, the roots were cut into approximately 1 cm long fragments and placed in Petri dishes containing ½ strength PDA medium and incubated under alternating cycles of 12 h BLB fluorescent light and 12 h darkness. After 10 to 15 days of incubation, fungal colonies developing from the root pieces were sub-cultured separately in Petri dishes containing PDA and SNA agar, incubated as described previously and identified based on cultural characteristics and microscopic examination of conidiogenous cells^4^.

The response of the tested legume species to *F*. *pisi* was determined according to criteria adopted in a slightly modified form from Kolander *et al*.^14^. The accessions were considered symptomatic hosts if the inoculated isolate was re-isolated from surface sterilized roots, the average disease severity rating was higher than 2 and significantly greater than in un-inoculated control plants. Accessions were also considered symptomatic if DSR < 2 but there was a significant reduction in mean plant biomass of inoculated treatment compared to the corresponding control. Some of the control plants showed moderate symptoms on the roots (mean DSR > 2) caused by factors other than inoculation with *F*. *pisi*, and in this case the host was considered susceptible if the final disease severity level of inoculated treatments was significantly higher than that of the corresponding control plants. The accessions were considered asymptomatic hosts if disease rating was less than or equal to 2, there was no significant reduction in biomass, and the fungus was re-isolated from the root parts following surface sterilization.

### Data analysis

All statistical analyses were done using R statistical software^45^ (version 3.3.1). For aggressiveness assay of selected FSSC isolates to pea, the normality of data distribution and homogeneity of variances were tested by Shapiro-Wilks-W-Test and Levene’s test, respectively. Prior to statistical analysis, disease severity index (DI) values were square root transformed. The data were first subjected to one way ANOVA to analyze differences in mean effects of different phylogenetic groups on root rot severity and plant biomass. Mean separations were made by Tukey HSD test. Differences among single isolates were tested separately by comparing means from inoculated treatments (each isolate) and un-inoculated control using Dunnett’s t test^46^. Treatments were considered significantly different if *P* ≤ 0.05.

Due unequal variances of many groups, the data on the host range of *Fusarium pisi* were subjected to nonparametric analyses. Differences in root rot severity ratings and biomass of inoculated treatments and corresponding un-inoculated controls were compared with 2 by 2 comparisons using the non-parametric ranking procedure of the Dunn’s test^47^.

### Data availability

The partial translation elongation factor 1-alpha (tef1) and the second-largest subunit of RNA polymerase II (rpb2) sequences of all strains used in this study were submitted to GenBank database. All fungal and plant materials are maintened at University of Kassel and are available for resesearch purposes upon request (representative fungal strains were deposited in the culture collection of the CBS-KNAW Fungal Biodiversity Centre, Utrecht, The Netherlands).
